# Efficacy of polyethylene glycol loxenatide versus insulin glargine on glycemic control in patients with type 2 diabetes: a randomized, open-label, parallel-group trial

**DOI:** 10.3389/fphar.2023.1171399

**Published:** 2023-05-04

**Authors:** Shuo Zhang, Chuanyan Zhang, Jingxian Chen, Feiying Deng, Zezhen Wu, Dan Zhu, Fengwu Chen, Yale Duan, Yue Zhao, Kaijian Hou

**Affiliations:** ^1^ School of Public Health, Shantou University, Shantou, China; ^2^ Shantou University Medical College, Shantou, China; ^3^ Department of Endocrine and Metabolic Diseases, Longhu People’s Hospital, The First Affiliated Hospital of Shantou University Medical College, Shantou, China; ^4^ Department of Medical Affairs, Jiangsu Hansoh Pharmaceutical Group Co., Ltd., Shanghai, China

**Keywords:** polyethylene glycol loxenatide, continuous glucose monitoring, time-in-range, type 2 diabetes, glucagon-like peptide 1 receptor agonist, insulin glargine

## Abstract

**Objective:** This trial aimed to evaluate the glycemic control of polyethylene glycol loxenatide measured with continuous glucose monitoring (CGM) in patients with type 2 diabetes mellitus (T2DM), with the hypothesis that participants given PEG-Loxe would spend more time in time-in-range (TIR) than participants were given insulin glargine after 24 weeks of treatment.

**Methods:** This 24-week, randomized, open-label, parallel-group study was conducted in the Department of Endocrine and Metabolic Diseases, Longhu Hospital, Shantou, China. Participants with T2DM, who were ≥45 years of age, HbA1c of 7.0%–11.0%, and treated at least 3 months with metformin were randomized (1:1) to receive PEG-Loxe or insulin glargine. The primary endpoint was TIR (blood glucose range: 3.9–10.0 mmol/L) during the last 2 weeks of treatment (weeks 22–24).

**Results:** From March 2020 to April 2022, a total of 107 participants with T2DM were screened, of whom 78 were enrolled into the trial (*n* = 39 per group). At the end of treatment (weeks 22–24), participants given PEG-Loxe had a greater proportion of time in TIR compared with participants given insulin glargine [estimated treatment difference (ETD) of 13.4% (95% CI, 6.8 to 20.0, *p* < 0.001)]. The tight TIR (3.9–7.8 mmol/L) was greater with PEG-Loxe *versus* insulin glargine, with an ETD of 15.6% (95% CI, 8.9 to 22.4, *p* < 0.001). The time above range (TAR) was significantly lower with PEG-Loxe *versus* insulin glargine [ETD for level 1: −10.5% (95% CI: −14.9 to −6.0), *p* < 0.001; ETD for level 2: −4.7% (95% CI: −7.9 to −1.5), *p* = 0.004]. The time below range (TBR) was similar between the two groups. The mean glucose was lower with PEG-Loxe *versus* insulin glargine, with an ETD of −1.2 mmol/L (95% CI, −1.9 to −0.5, *p* = 0.001). The SD of CGM glucose levels was 1.88 mmol/L for PEG-Loxe and 2.22 mmol/L for insulin glargine [ETD -0.34 mmol/L (95% CI: −0.55 to −0.12), *p* = 0.002], with a similar CV between the two groups.

**Conclusion:** The addition of once-weekly GLP-1RA PEG-Loxe to metformin was superior to insulin glargine in improving glycemic control and glycemic variability evaluated by CGM in middle-aged and elderly patients with T2DM.

## 1 Introduction

The prevalence of type 2 diabetes mellitus (T2DM) continues to rise globally and shows no signs of stabilizing. The global prevalence of T2DM was reported to be 6.28% (462 million individuals) in 2017 (Khan et al., 2020). Recently released data estimates that the number of individuals with T2DM worldwide will increase to 700 million by 2045 (Sun et al., 2021). T2DM is considered a typical age-related disease and usually manifests after the age of 40 (Jung et al., 2022). The incidence of T2DM rises with age, with pooled global estimates suggesting that it is most commonly seen around 55–59 years of age (Tinajero and Malik, 2021).

Glucagon-like peptide-1 (GLP-1) receptor agonist (GLP-1RA) is a new class of glucose-lowering medications that exert efficacy by promoting insulin secretion and inhibiting glucagon secretion. GLP-1RA drugs also have multiple benefits, including weight loss, improvement of lipid profile, and cardiovascular protection (Pedrosa et al., 2022).

Polyethylene glycol loxenatide (PEG-Loxe) is a once-weekly long-acting GLP-1RA, derived from the structural modification of exendin-4 (Cai et al., 2023). The efficacy and safety of PEG-Loxe has been established in patients with T2DM. In a phase 3a trial, PEG-Loxe at 0.2 mg led to a 1.34% reduction in glycated hemoglobin (HbA1c) after 24 weeks of treatment (Shuai et al., 2021). In the phase 3b trial, PEG-Loxe at 0.2 mg combined with metformin demonstrated a similar HbA1c reduction (−1.14%) (Gao et al., 2020). Moreover, our previous study reported that PEG-Loxe improved endothelial cell function in middle-aged and elderly patients with T2DM (Chen et al., 2022). Safety data showed that PEG-Loxe was well tolerated, with <3% of hypoglycemic events, <2% of antidrug antibodies, and 10%–25% of patients experiencing gastrointestinal disorders (Gao et al., 2020; Shuai et al., 2021).

**FIGURE 1:**
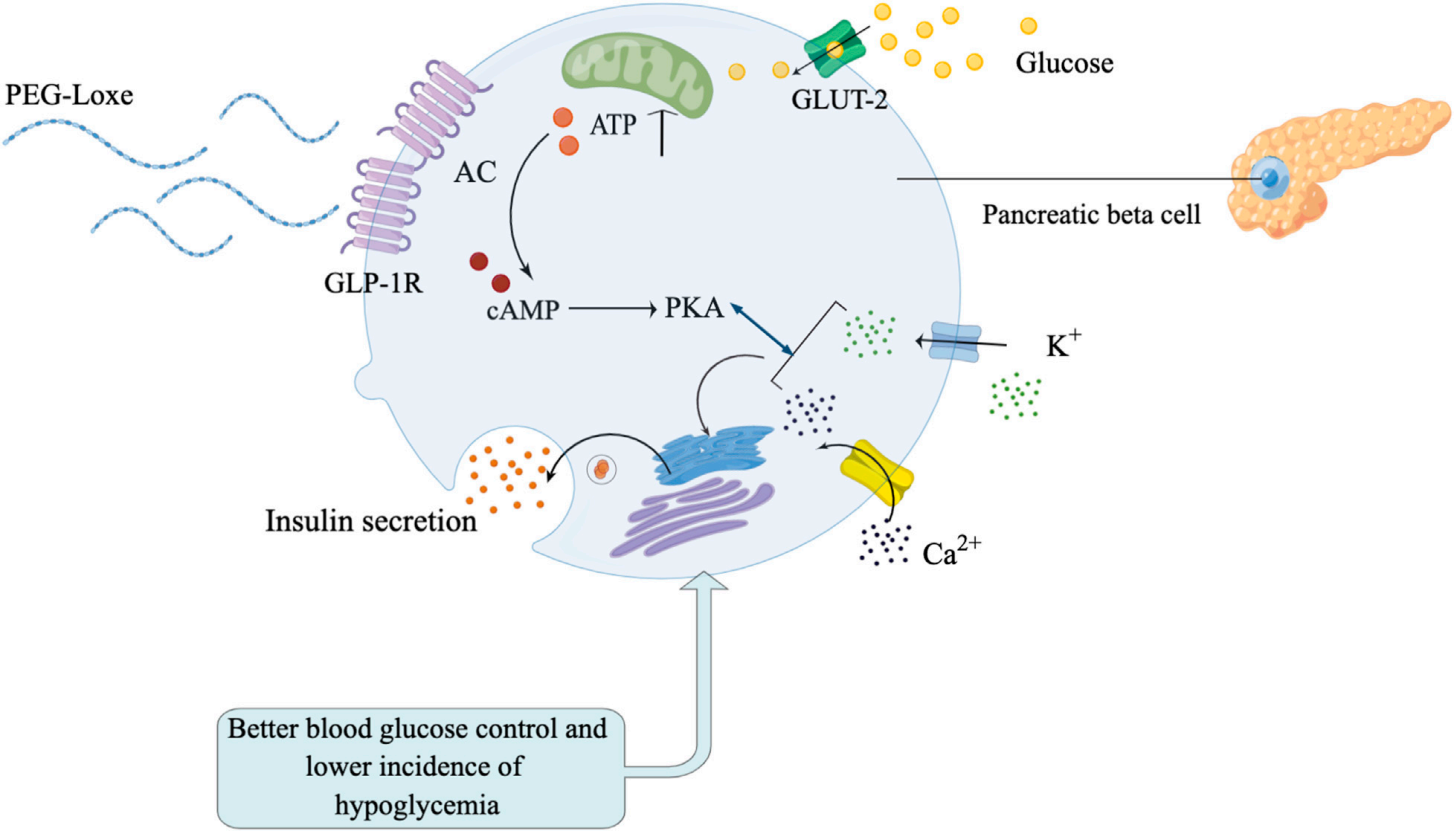
Polyethylene glycol loxenatide (PEG-Loxe) can effectively stimulate pancreatic beta cells to secrete insulin to control blood glucose, and there are fewer adverse reactions such as hypoglycemia.

HbA1c is the gold standard for glycemic monitoring in patients with T2DM. However, HbA1c only reflects glycemic status for the prior 3 months, and cannot reflect short-term glycemic variability and hypoglycemia risk (Hempe and Hsia, 2022). Recently, the use of continuous glucose monitoring (CGM) technology has been increasing, providing a range of new indicators [time-in-range (TIR), coefficient of variation (CV)] in glycemic monitoring. Among them, TIR is an important indicator for evaluating glycemic control, and studies have shown that TIR can predict the risk of diabetes complications (Advani, 2020; Yoo and Kim, 2020). The mechanism of PEG-Loxe lowering blood glucose is shown in Figure 1. Recently published studies reported that GLP-1RA (liraglutide, semaglutide, and dulaglutide) significantly improved TIR in patients with T2DM (Sofizadeh et al., 2019; Wang et al., 2019; Al Hayek and Al Dawish, 2022). However, it is unclear whether PEG-Loxe influences TIR in patients with T2DM.

This trial aimed to evaluate the glucose control measured with CGM in participants with T2DM, based on the hypothesis that participants given PEG-Loxe would spend more time in TIR (3.9–10.0 mmol/L) than participants were given insulin glargine after 24 weeks of treatment. To test the hypothesis, a 24-week, randomized, open-label, parallel-group trial was conducted comparing PEG-Loxe vs. insulin glargine in middle-aged and elderly patients with T2DM. The primary endpoint was TIR, with secondary endpoints including tight TIR, time above range (TAR), time below range (TBR), mean glucose, glycemic variability and HbA1c.

## 2 Materials and methods

### 2.1 Trial design and participants

**FIGURE 2:**
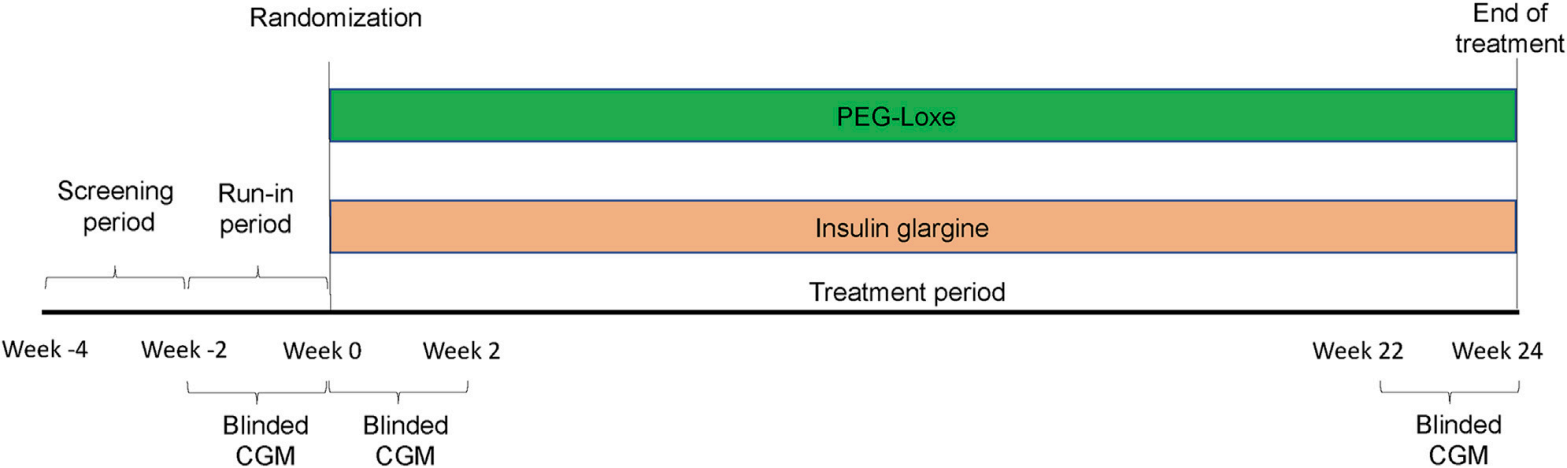
This study is consisted of three periods: a screening period (2 weeks), a run-in period (2 weeks), and a treatment period (24 weeks).

This 24-week, randomized, open-label, parallel-group trial was conducted between March 2020 and October 2022 by the Department of Endocrine and Metabolic Diseases, Longhu Hospital, Shantou, China. The Ethics Committee of Longhu Hospital approved the trial protocol (No. LHLL2019002), which followed local regulations and the Declaration of Helsinki. All patients provided written informed consent. This clinical trial was registered with ClinicalTrials.gov (ChiCTR1900026514) and consisted of three periods: a screening period (2 weeks), a run-in period (2 weeks), and a treatment period (24 weeks) (Figure 2).

Key inclusion criteria included patients with T2DM (according to the 2020 China guideline criteria) (Society, 2021), ≥45 years of age, HbA1c of 7.0%–11.0%, and treated with metformin at least 3 months. Key exclusion criteria included patients with severe diabetic complications, gastrointestinal disease or surgical history, and history of any cardiovascular diseases that had occurred within the last 3 months. The entire inclusion and exclusion criteria are displayed in Supplementary Figure S1.

### 2.2 Randomization and masking

Eligible participants were randomly assigned (1:1) to receive once-weekly PEG-Loxe (0.2 mg) or once-daily insulin glargine (100 U/mL) by using the Interactive Web Response System. Participants were stratified according to baseline HbA1c (≤8.5% or >8.5%). An open-label design was used in this trial, mainly due to differences in administration frequency and injection device between the two groups.

### 2.3 Procedures

During the run-in period, participants received training for self-injection and the use of the CGM system (Abbott FreeStyle Libre Pro, California, United States). At the start of treatment (Week 0), participants received PEG-Loxe or insulin glargine as an add-on to metformin treatment. PEG-Loxe (Hansoh Pharma) was administered subcutaneously, once weekly at a dose of 0.2 mg. Insulin glargine 100 U/mL (Sanofi) was administered subcutaneously once daily. Based on baseline fasting glucose, the initial dose of insulin glargine was at least 8 U per day. The insulin dose was adjusted twice a week, following a treat-to-target algorithm, based on the previous three self-monitoring of blood glucose (SMBG) values (Wang et al., 2019).

Prior to randomization, general information was collected, including demographic data, vital signs, and medical history. Blood was drawn for HbA1c testing.

The CGM data were collected during three CGM measuring periods, namely, run-in period (weeks -2–0), the first 2 weeks of treatment (weeks 0–2), and the last 2 weeks of treatment (weeks 22–24). The interstitial glucose values were measured at 15 min intervals by the CGM system (Goldenberg et al., 2021). To be eligible for the randomization, participants needed to have at least 235 h (equivalent to 9.8 days) of available CGM data. The CGM system was removed before randomization. The process was repeated on two occasions at the start of treatment (weeks 0–2), and the end of treatment (weeks 22–24). The CGM readings were blinded to participants but were recorded by the device.

### 2.4 Endpoints

The primary endpoint was TIR, the percentage of time spent in target range (3.9–10.0 mmol/L) during the last 2 weeks of treatment (weeks 22–24), as recommended from the International CGM Consensus (2019) (Battelino et al., 2019). The secondary endpoints included tight TIR [the percentage of time spent in tight target range (3.9–7.8 mmol/L)], TAR (level 1: 10.1–13.9 mmol/L; level 2: >13.9 mmol/L), TBR (level 1: 3.0–3.8 mmol/L; level 2: <3.0 mmol/L), mean glucose, and glycemic variability [as measured by within-day standard deviation (SD) and CV] during the last 2 weeks of treatment (weeks 22–24); and changes in HbA1c at 24 weeks. The exploratory endpoints included TIR, tight TIR, TAR, TBR, mean glucose, and glycemic variability during the first 2 weeks of treatment (weeks 0–2).

Because the safety profiles of PEG-Loxe and insulin glargine are well characterized, reportable adverse events (AEs) were limited to serious AEs (SAEs), AEs leading to discontinuation of trial drugs, and severe hypoglycemia (requiring assistance from another person).

### 2.6 Statistical analyses

The planned sample size of 78 participants was randomized 1:1 to receive either PEG-Loxe or insulin glargine. This sample size would provide 90% power to detect an absolute difference of ≥25% in TIR (assuming an SD of 30%, a type I error of 5%, and a 20% withdrawal rate) (Jendle et al., 2016) between the PEG-Loxe and insulin glargine groups.

The continuous variables were examined for normal distribution using Kolmogorov-Smirnov test. Baseline characteristics were evaluated using independent samples *t*-test, Mann-Whitney *U* test, or χ^2^ test. The analyses of primary endpoints were performed for the full analysis set (participants exposed to ≥1 treatment and had a baseline TIR), and a mixed model for repeated measurements (MMRM) was used, with a multiple linear imputation method (Rubin, 1987). Fixed effects included group, time, and interactions between group and time. Covariates included baseline TIR and sex. The analyses of secondary endpoints and exploratory endpoints were performed for the per-protocol set (participants who completed the study without any major protocol violations). A similar model was implemented to analyze tight TIR, TAR, TBR, mean glucose, SD, and CV. The χ^2^ test or Fisher exact test was used to analyze categorical variables. Sensitivity analyses were performed for the primary endpoint, based on the per-protocol set.

Analyses were performed using Statistical Analysis System (SAS) version 9.4. A *p*-value <0.05 (2-sided) was considered statistically significant.

## 3 Results

**FIGURE 3:**
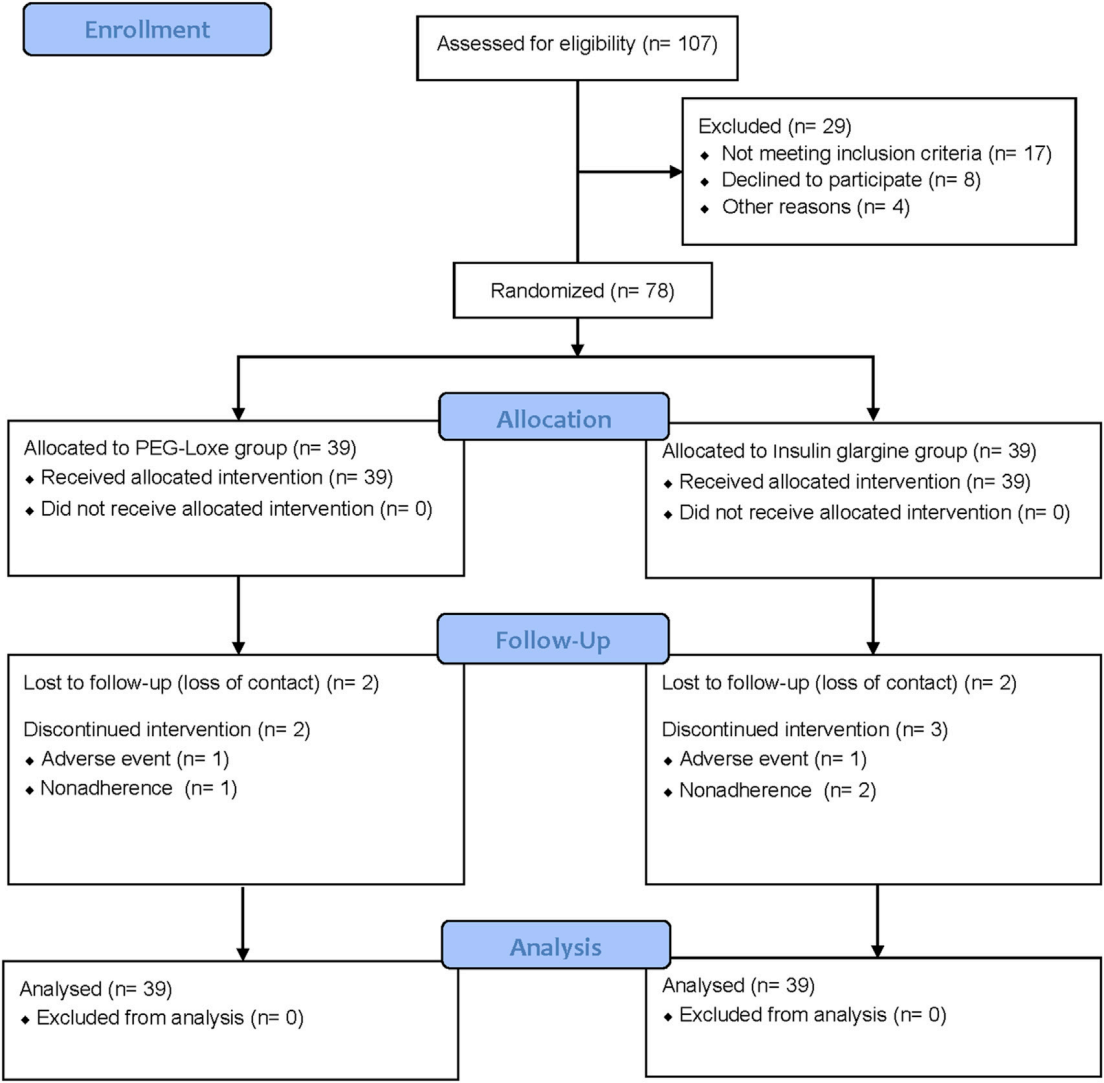
Consort flow diagram.

Between March 2020 and April 2022, a total of 107 adults with T2DM were screened, of whom 78 were enrolled into the trial (*n* = 39 per group). Of the enrolled, 69 participants (88.5%) completed the trial (*n* = 35 and n = 34 for PEG-Loxe and insulin glargine, respectively) (Figure 3). The baseline characteristics were similar between the two groups (Table 1).

**TABLE 1:** Baseline characteristics of participants.

	PEG-loxe (n = 39)	Insulin glargine (n = 39)	*p*-Value
Women, N (%)	17 (43.6)	22 (56.4)	0.26
Age, y	68.3 (10.4)	67.4 (10.2)	0.73
Duration, y	10.6 (7.6)	9.9 (5.7)	0.67
Body weight, kg	59.4 (12.6)	59.9 (7.6)	0.82
BMI, kg/m2	22.7 (3.1)	22.6 (2.4)	0.85
HbA1c, %	8.56 (1.27)	8.79 (1.10)	0.39
CGM			
Mean glucose, mmol/L	10.6 (2.3)	10.1 (2.3)	0.33
TIR (3.9–10.0 mmol/L), %	48.6 (26.8)	52.6 (23.7)	0.48
Tight TIR (3.9–7.8 mmol/L), %	32.1 (19.0)	36.4 (18.1)	0.31
TAR (>10.0 mmol/L), %	50.8 (27.0)	45.8 (25.2)	0.40
TAR (level 1: 10.1–13.9 mmol/L), %	29.6 (15.7)	26.7 (16.3)	0.45
TAR (level 2: >13.9 mmol/L), %	21.2 (0.4)	19.1 (0.9)	0.42
TBR (<3.9 mmol/L), %	0.0 (0.0, 1.0)	0.0 (0.0, 2.0)	0.67
TBR (level 1: 3.0–3.8 mmol/L), %	0.0 (0.0, 1.0)	0.0 (0.0, 2.0)	0.70
TBR (level 2: <3.0 mmol/L), %	0.0 (0.0, 0.0)	0.0 (0.0, 0.0)	0.30

Abbreviations: BMI, body mass index; HbA1c, glycated hemoglobin; CGM, continuous glucose monitoring; TIR, time in range; Tight TIR, tight time in range; TAR, time above range; TBR, time below range. Data are expressed as mean (SD) or median (interquartile range), unless otherwise indicated.

### 3.1 Primary endpoints

During the last 2 weeks of treatment, participants given PEG-Loxe spent significantly more TIR (3.9–10.0 mmol/L) within the 2-week period than did those given insulin glargine. The least-square mean (LSM) TIR was 81.4% (95% CI, 76.7–86.0) for the PEG-Loxe group and 67.9% (95% CI, 63.3–72.6) for the insulin glargine group, with an estimated treatment difference (ETD) of 13.4% (95% CI, 6.8 to 20.0, *p* < 0.001) (Table 2). This was equivalent to an additional 3.22 h of TIR per day with PEG-Loxe *versus* insulin glargine. Sensitivity analyses for the primary endpoint showed similar results (Supplementary Table S1).

**TABLE 2:** Primary and secondary endpoints at the end of treatment (weeks 22–24).

	PEG-loxe mean (95% CI)	Insulin glargine mean (95% CI)	ETD (PEG-Loxe− insulin glargine) (95% CI)	*p*-Value
Primary endpoint	n = 39	n = 39		
TIR (3.9–10.0 mmol/L), %	81.4 (76.7, 86.0)	67.9 (63.3, 72.6)	13.4 (6.8, 20.0)	<0.001
Secondary endpoints	n = 35	n = 34		
Tight TIR (3.9–7.8 mmol/L), %	67.3 (62.6, 72.1)	51.7 (46.8, 56.5)	15.6 (8.9, 22.4)	<0.001
TAR (>10.0 mmol/L), %	17.7 (12.7, 22.7)	33.1 (28.0, 38.2)	−15.4 (−22.5, −8.3)	<0.001
TAR (level 1: 10.1–13.9 mmol/L), %	12.1 (9.0, 15.2)	22.6 (19.4, 25.8)	−10.5 (−14.9, −6.0)	<0.001
TAR (level 2: >13.9 mmol/L), %	5.7 (3.5, 8.0)	10.4 (8.1, 12.7)	−4.7 (−7.9, −1.5)	0.004
TBR (<3.9 mmol/L), %	1.7 (0.8, 2.7)	1.1 (0.1, 2.0)	0.7 (−0.7, 2.0)	0.32
TBR (level 1: 3.0–3.8 mmol/L), %	1.5 (0.7, 2.2)	1.0 (0.3, 1.7)	0.5 (−0.5, 1.5)	0.36
TBR (level 2: <3.0 mmol/L), %	0.3 (0.1, 0.5)	0.1 (−0.2, 0.3)	0.2 (−0.1, 0.5)	0.25
Mean glucose, mmol/L	7.8 (7.3, 8.3)	9.0 (8.5, 9.5)	−1.2 (−1.9, −0.5)	0.001
Change in HbA1c, %	−1.1 (−1.4, −0.8)	−0.9 (−1.2, −0.6)	−0.2 (−0.6, 0.2)	0.31
SD, mmol/L	1.88 (1.73, 2.03)	2.22 (2.07, 2.37)	−0.34 (−0.55, −0.12)	0.002
CV, %	24.0 (22.7, 25.2)	23.9 (22.6, 25.2)	0.1 (−1.7, −1.9)	0.94

Abbreviations: TIR, time in range; Tight TIR, tight time in range; TAR, time above range; TBR, time below range; HbA1c, glycated hemoglobin; SD, standard deviation; CV, coefficient of variation.

### 3.2 Secondary endpoints

At the end of treatment (weeks 22–24), the LSM (95% CI) tight TIR (3.9–7.8 mmol/L) was greater with PEG-Loxe *versus* insulin glargine, with an ETD of 15.6% (95% CI, 8.9 to 22.4, *p* < 0.001). The TAR (level 1: 10.1–13.9 mmol/L, and level 2: >13.9 mmol/L) were significantly lower with PEG-Loxe *versus* insulin glargine [ETD for level 1: −10.5% (95% CI: −14.9 to −6.0), *p* < 0.001; ETD for level 2: −4.7% (95% CI: −7.9 to −1.5), *p* = 0.004]. The TBR (level 1: 3.0–3.8 mmol/L, and level 2: <3.0 mmol/L) was similar between the two groups. The mean glucose was lower with PEG-Loxe *versus* insulin glargine, with an ETD of −1.2 mmol/L (95% CI, −1.9 to −0.5, *p* = 0.001). The changes in HbA1c at 24 weeks were similar with PEG-Loxe [95% CI, −1.1% (−1.4 to −0.8)] and insulin glargine [-0.9% (−1.2to −0.6), *p* = 0.31]. The SD of CGM glucose levels was 1.88 mmol/L for PEG-Loxe and 2.22 mmol/L for insulin glargine [ETD -0.34 mmol/L (95% CI: −0.55 to −0.12), *p* = 0.002], with a similar CV between the two groups (Table 2). Supplementary Figure S2 shows CGM profiles examples of 2 participants during the last 2 weeks of treatment (weeks 22–24).

### 3.3 Exploratory endpoints

During the first 2 weeks of treatment (weeks 0–2), the LSM (95% CI) TIR (3.9–10.0 mmol/L) was similar with PEG-Loxe [55.6% (52.0–59.2)] and insulin glargine [54.5% (50.7–58.2), *p* = 0.66]. The tight TIR, TAR, TBR, mean glucose, SD, and CV were similar between the two groups (Table 3).

**TABLE 3:** Exploratory endpoints during the first 2 weeks of treatment.

	PEG-loxe (n = 35) mean (95% CI)	Insulin glargine (n = 34) mean (95% CI)	ETD (PEG-Loxe− insulin glargine) (95% CI)	*p*-Value
TIR (3.9–10.0 mmol/L), %	55.6 (52.0, 59.2)	54.5 (50.7, 58.2)	1.2 (−4.1, 6.4)	0.66
Tight TIR (3.9–7.8 mmol/L), %	33.5 (29.8, 37.1)	32.1 (28.4, 35.8)	1.3 (−3.9, 6.5)	0.61
TAR (>10.0 mmol/L), %	42.5 (38.8, 46.3)	44.2 (40.3, 48.1)	−1.7 (−7.1, 3.8)	0.55
TAR (level 1: 10.1–13.9 mmol/L), %	25.2 (22.9, 27.6)	26.3 (23.9, 28.8)	−1.1 (−4.5, 2.3)	0.52
TAR (level 2: >13.9 mmol/L), %	17.4 (15.8, 19.1)	17.9 (16.1, 19.6)	−0.4 (−2.9, 2.0)	0.73
TBR (<3.9 mmol/L), %	1.5 (0.8, 2.2)	1.4 (0.7, 2.2)	0.1 (−1.0, 1.1)	0.91
TBR (level 1: 3.0–3.8 mmol/L), %	1.2 (0.7, 1.8)	1.2 (0.6, 1.7)	0.1 (−0.7, 0.9)	0.83
TBR (level 2: <3.0 mmol/L), %	0.2 (0.1, 0.4)	0.3 (0.1, 0.5)	−0.0 (−0.3, 0.2)	0.83
Mean glucose, mmol/L	9.9 (9.6, 10.3)	10.0 (9.7, 10.4)	−0.1 (−0.6, 0.4)	0.65
SD, mmol/L	2.13 (2.02, 2.25)	2.16 (2.05, 2.28)	−0.03 (−0.20, 0.13)	0.71
CV, %	21.9 (21.0, 22.9)	22.1 (21.2, 23.1)	−0.22 (−1.6, 1.2)	0.76

Abbreviations: TIR, time in range; Tight TIR, tight time in range; TAR, time above range; TBR, time below range; SD, standard deviation; CV, coefficient of variation.

### 3.4 Safety

The incidence of SAEs was similar with PEG-Loxe and insulin glargine. Two (2.6%) patients (PEG-Loxe: N = 1 [2.6%] and insulin glargine: N = 1 [2.6%]) discontinued treatment because of AEs. The incidence of severe hypoglycemia was 0.0% (0/39) and 2.6% (1/39) in the PEG-Loxe and insulin glargine groups, respectively. No new safety issues related to PEG-Loxe were identified (Supplementary Table S2).

## 4 Discussion

This is the first head-to-head study designed to evaluate the efficacy of PEG-Loxe *versus* insulin glargine on glycemic control measured by CGM in middle-aged and elderly patients with T2DM. In the present trial, compared with insulin glargine, the addition of PEG-Loxe with metformin resulted in significantly greater TIR (3.9–10.0 mmol/L) during the last 2 weeks of treatment. This was accompanied by similar HbA1c reduction between the two groups.

A small fluctuation in plasma drug concentrations is one of the advantages of long-acting GLP-1RAs (Gentilella et al., 2019), which may indicate that long-acting GLP-1RAs exert stable glucose-lowering effects. A few CGM studies have been done on the effects of GLP-1RA on glucose fluctuation. Dulaglutide treatment resulted significantly greater improvement in TIR (from 40.7% to 83.1%) compared with insulin glargine (from 52.2% to 73.0%) in patients with T2DM at 26 weeks (Jendle et al., 2016). TIR increased significantly in liraglutide-treated patients, from 57% at baseline to 75% at 24 weeks (Lane et al., 2014). As a long-acting GLP-1RA, PEG-Loxe consists of modified exendin-4 covalently linked to a branched polyethylene glycol (PEG) (Chen et al., 2017), thereby enhancing resistance to proteolysis and reducing renal clearance, which increases stability and prolongs protein circulating times (Kang et al., 2009). In the previous pharmacokinetic trial, the concentration–time profile of PEG-Loxe showed small peak-to-trough fluctuation, indicating that plasma concentration of PEG-Loxe was relatively stable (Yang et al., 2015). This suggests that PEG-Loxe may exert stable glucose-lowering effects. In this study, PEG-Loxe displayed similar potency for TIR improvement (from 48.6% to 81.4%) as dulaglutide. Moreover, insulin glargine treatment resulted in a TIR level of 67.9% in this study, which is comparable to previous reports in which insulin glargine increased TIR level to 56.0%–70.7% (Wang et al., 2019; Goldenberg et al., 2021).

The significantly reduced hyperglycemia (as measured by TAR) in the PEG-Loxe group supported a significant improvement in postprandial glycemic control with PEG-Loxe. This improvement was also seen in 6-point SMBG profiles of a previous phase 3a study (Shuai et al., 2021). The fluctuations of postprandial glucose are more pronounced in Asian patients with T2DM, thus postprandial glycemic control is particularly important for Asian patients with T2DM (Lu, 2019). The GLP-1RAs improve both fasting and postprandial glucose through multiple mechanisms, including promoting insulin secretion, inhibiting glucagon secretion, slowing gastric emptying, and reducing appetite (Shaefer et al., 2015). Among these, slowing gastric emptying and reducing appetite contribute to postprandial glycemic control (Triplitt and Solis-Herrera, 2015; Lu, 2019).

Recent evidence has revealed the role of glycemic variability (as measured by SD and CV) in the development of diabetes complications (Rodbard, 2018; Umpierrez, , 2018). Glycemic variability is an important consideration in evaluating the quality of glycemic control. The SD level is moderately correlated with mean glucose and almost unrelated to hypoglycemic risk (Rodbard, 2018). In the present study, patients given PEG-Loxe showed significantly lower SD than those given insulin glargine, reflecting mean glucose compared with insulin glargine (7.8 mmol/L and 9.0 mmol/L, respectively). The CV is highly correlated with hypoglycemia risk (Rodbard, 2018). According to the International CGM Consensus (2019), patients should strive for ≤36% of CV (Battelino et al., 2019). In the present study, the CV were similar in the PEG-Loxe and insulin glargine groups (24.0% and 23.9%, respectively), and both met the recommendation of the International CGM Consensus (2019). This is also consistent with the results of hypoglycemia (as measured by TBR) in this study.

The onset of action of insulin glargine occurred 1 h after subcutaneous administration (Rosskamp and Park, 1999). In an unpublished phase one study, PEG-Loxe lowered blood glucose levels in patients with T2DM 1 h after subcutaneous injection. In this study, we explored the efficacy of PEG-Loxe and insulin glargine in the initial stage of treatment (weeks 0–2). The results showed similar efficacy between the two groups. This suggests that PEG-Loxe is similar to insulin glargine and can take effect in a short time.

One limitation of this study is the small sample size. Another limitation is the open-label design, which might increase the risk of bias. Large scale and longer duration studies are needed to further validate the benefits of PEG-Loxe.

In conclusion, the addition of once-weekly GLP-1RA PEG-Loxe to metformin was superior to insulin glargine in improving glycemic control and glycemic variability evaluated by CGM in middle-aged and elderly patients with T2DM.

## Data Availability

The raw data supporting the conclusion of this article will be made available by the authors, without undue reservation.
