# Supplementary material for: Efficacy of polyethylene glycol loxenatide versus insulin glargine on glycemic control in patients with type 2 diabetes: a randomized, open-label, parallel-group trial
Source: Front Pharmacol. 2023 May 4;14:1171399. doi: 10.3389/fphar.2023.1171399 (PMC10194654; doi:10.3389/fphar.2023.1171399)
Supplement: Supplementary file 1 [file Table1.DOCX]

Supplementary Material

Figure S1





Figure S1 | The inclusion and exclusion criteria.

Table S1 | Sensitivity analyses of the primary endpoint at week 24 (per-protocol set).

|  | PEG-Loxe | |  |  | Insulin glargin | |  |  |
| --- | --- | --- | --- | --- | --- | --- | --- | --- |
|  | No. of  patients | Mean  (95% CI) |  |  | No. of  patients | Mean  (95% CI) | ETD (PEG-Loxe− Insulin glargine)  (95% CI) | *P* value |
| TIR (3.9–10.0 mmol/L), % | 35 | 80.5 (75.7, 85.2) |  |  | 34 | 65.9 (61.1, 70.8) | 14.5 (7.7, 21.4) | ＜0.001 |

Table S2 | Summary of Safety

|  | PEG-Loxe  No. (%)  (n=39) | Insulin glargin  No. (%)  (n=39) |
| --- | --- | --- |
| Any SAE |  |  |
| Death | 0 (0) | 0 (0) |
| Other | 2 (5.1) | 3 (7.7) |
| Discontinuation because of AEs | 1 (2.6) | 1 (2.6) |
| Severe hypoglycemia | 0 (0) | 1 (2.6) |
