# Supplementary figures and images for: Efficacy of polyethylene glycol loxenatide versus insulin glargine on glycemic control in patients with type 2 diabetes: a randomized, open-label, parallel-group trial
Source: Front Pharmacol. 2023 May 4;14:1171399. doi: 10.3389/fphar.2023.1171399 (PMC10194654; doi:10.3389/fphar.2023.1171399)

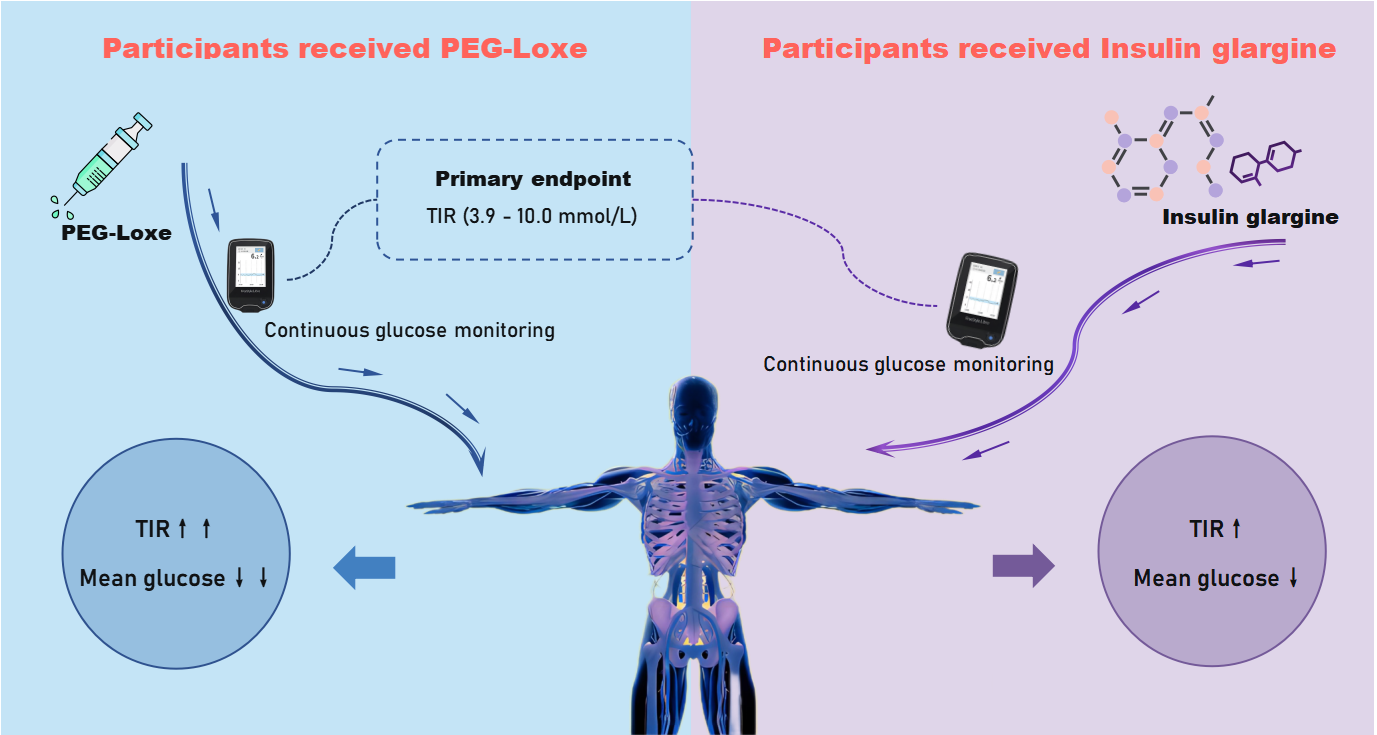

Supplement: Supplementary file 2 [file Image1.TIF]
